# 3-Amino­phenyl naphthalene-1-sulfonate

**DOI:** 10.1107/S1600536808041032

**Published:** 2008-12-10

**Authors:** Jasmine P. Vennila, Helen P. Kavitha, D. John Thiruvadigal, B. R. Venkatraman, V. Manivannan

**Affiliations:** a Department of Physics, Panimalar Institute of Technology, Chennai 600 095, India; b Department of Chemistry, SRM University, Ramapuram, Chennai 600 089, India; c Department of Physics, SRM University, Kattankulathur Campus, Chennai, India; d Department of Chemistry, Periyar E.V.R. College, Tiruchirappalli 620 023, India; e Department of Physics, Presidency College, Chennai 600 005, India

## Abstract

In the title compound, C_16_H_13_NO_3_S, the plane of the naphthalene ring system forms a dihedral angle of 64.66 (10)° with the benzene ring. The mol­ecular structure is stabilized by weak intra­molecular C—H⋯O inter­actions and the crystal packing is stabilized by weak inter­molecular N—H⋯O and C—H⋯O inter­actions and by π–π stacking inter­actions of the inversion-related naphthalene units [centroid–centroid  distance  of 3.7373 (14) Å].

## Related literature

For the structures of closely related compounds, see: Manivannan *et al.* (2005*a* ▶,*b* ▶); Ramachandran *et al.*(2007 ▶); Vennila *et al.* (2008 ▶). For applications, see: Spungin *et al.* (1984 ▶); Yachi *et al.* (1989 ▶).
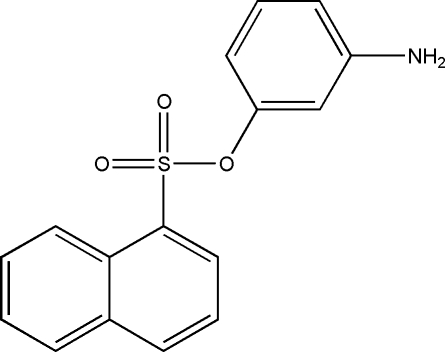


## Experimental

### 

#### Crystal data

C_16_H_13_NO_3_S
*M* *_r_* = 299.33
Monoclinic, 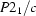

*a* = 8.4558 (2) Å
*b* = 8.6712 (3) Å
*c* = 19.5915 (6) Å
β = 100.321 (2)°
*V* = 1413.24 (7) Å^3^
*Z* = 4
Mo *K*α radiation
μ = 0.24 mm^−1^
*T* = 295 (2) K
0.30 × 0.25 × 0.20 mm

#### Data collection

Bruker Kappa APEXII diffractometer
Absorption correction: multi-scan (*SADABS*; Sheldrick, 1996 ▶) *T*_min_ = 0.932, *T*_max_ = 0.954
19808 measured reflections
4981 independent reflections
3126 reflections with *I* > 2σ(*I*)
*R*_int_ = 0.023

#### Refinement

*R*[*F* ^2^ > 2σ(*F* ^2^)] = 0.055
*wR*(*F* ^2^) = 0.174
*S* = 1.05
4981 reflections
190 parameters
H-atom parameters constrained
Δρ_max_ = 0.46 e Å^−3^
Δρ_min_ = −0.44 e Å^−3^

### 

Data collection: *APEX2* (Bruker, 2004 ▶); cell refinement: *SAINT* (Bruker, 2004 ▶); data reduction: *SAINT*; program(s) used to solve structure: *SHELXS97* (Sheldrick, 2008 ▶); program(s) used to refine structure: *SHELXL97* (Sheldrick, 2008 ▶); molecular graphics: *PLATON* (Spek, 2003 ▶); software used to prepare material for publication: *SHELXL97*.

## Supplementary Material

Crystal structure: contains datablocks I, global. DOI: 10.1107/S1600536808041032/gk2179sup1.cif

Structure factors: contains datablocks I. DOI: 10.1107/S1600536808041032/gk2179Isup2.hkl

Additional supplementary materials:  crystallographic information; 3D view; checkCIF report

## Figures and Tables

**Table 1:** Hydrogen-bond geometry (Å, °)

*D*—H⋯*A*	*D*—H	H⋯*A*	*D*⋯*A*	*D*—H⋯*A*
C2—H2⋯O2	0.93	2.41	2.829 (3)	107
C9—H9⋯O3	0.93	2.56	3.127 (3)	120
N1—H1*B*⋯O3^i^	0.86	2.43	3.246 (3)	158
C7—H7⋯O2^ii^	0.93	2.56	3.422 (3)	154

Symmetry codes: (i) 
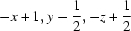
; (ii) 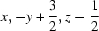.

**Fig. 1.**
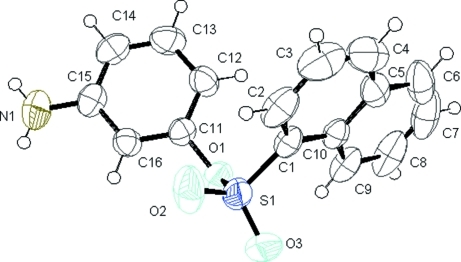
The molecular structure of the title compound, with atom labels and 50% probability displacement ellipsoids for non-H atoms.

**Fig. 2.**
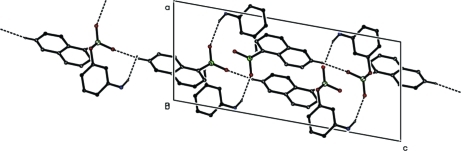
The packing viewed down the b axis. Hydrogen bonds are shown as dashed lines. H atoms not involved in hydrogen bonding have been omitted.

## supplementary crystallographic information

### Comment

Several compounds containing the *para*-toluene sulfonate moiety are used in the fields of biology and industry. The merging of lipids can be monitored using a derivative of *para*-toluene sulfonate (Yachi *et al.*, 1989). This method has been used in studying the membrane fusion during the acrosome reaction (Spungin *et al.*, 1984).

The plane of the benzene ring forms a dihedral angle of 64.66 (10) ° with the naphthalene ring system. The torsion angles O2—S1—C1—C2 and O3—S1—C1—C10 [5.58 (17) ° and 52.09 (16) °, respectively] indicate the *syn* conformation of sulfonyl moiety. The molecular structure is stabilized by weak intramolecular C—H···O interactions and the crystal packing is stabilized by weak intermolecular C—H···O interactions, N—H···O interactions and π-π stacking interactions of the naphthalene fragments related by inversion center

### Experimental

1-Napthalene sulfonyl chloride (5 mmol) dissolved in acetone (4 ml) was added dropwise to 3-amino phenol (5 mmol) in aqueous NaOH (4 ml, 5%) with constant shaking. The precipitated compound (3 mmol, yield 60%) was recrystlized from ethanol to get diffraction quality brown colored crystals.

### Refinement

H atoms were positioned geometrically and refined using riding model with C—H = 0.93 Å and *U*_iso_(H) = 1.2Ueq(C) for aromatic C—H and N—H = 0.86 Å and *U*_iso_(H) = 1.2Ueq(N) for N—H.

### Crystal data

**Table:**

C_16_H_13_NO_3_S	*F*(000) = 624
*M**_r_* = 299.33	*D*_x_ = 1.407 Mg m^−^^3^
Monoclinic, *P*2_1_/*c*	Mo *K*α radiation, λ = 0.71073 Å
Hall symbol: -P 2ybc	Cell parameters from 4818 reflections
*a* = 8.4558 (2) Å	θ = 2.2–25.4°
*b* = 8.6712 (3) Å	µ = 0.24 mm^−^^1^
*c* = 19.5915 (6) Å	*T* = 295 K
β = 100.321 (2)°	Block, brown
*V* = 1413.24 (7) Å^3^	0.30 × 0.25 × 0.20 mm
*Z* = 4	

### Data collection

**Table:**

Bruker Kappa APEX2 diffractometer	4981 independent reflections
Radiation source: fine-focus sealed tube	3126 reflections with *I* > 2σ(*I*)
graphite	*R*_int_ = 0.023
ω and φ scans	θ_max_ = 32.2°, θ_min_ = 2.1°
Absorption correction: multi-scan (*SADABS*; Sheldrick, 1996)	*h* = −12→10
*T*_min_ = 0.932, *T*_max_ = 0.954	*k* = −12→11
19808 measured reflections	*l* = −21→29

### Refinement

**Table:**

Refinement on *F*^2^	Primary atom site location: structure-invariant direct methods
Least-squares matrix: full	Secondary atom site location: difference Fourier map
*R*[*F*^2^ > 2σ(*F*^2^)] = 0.055	Hydrogen site location: inferred from neighbouring sites
*wR*(*F*^2^) = 0.174	H-atom parameters constrained
*S* = 1.05	*w* = 1/[σ^2^(*F*_o_^2^) + (0.0763*P*)^2^ + 0.3485*P*] where *P* = (*F*_o_^2^ + 2*F*_c_^2^)/3
4981 reflections	(Δ/σ)_max_ < 0.001
190 parameters	Δρ_max_ = 0.46 e Å^−^^3^
0 restraints	Δρ_min_ = −0.44 e Å^−^^3^

### Special details

**Table:**

Geometry. All e.s.d.'s (except the e.s.d. in the dihedral angle between two l.s. planes) are estimated using the full covariance matrix. The cell e.s.d.'s are taken into account individually in the estimation of e.s.d.'s in distances, angles and torsion angles; correlations between e.s.d.'s in cell parameters are only used when they are defined by crystal symmetry. An approximate (isotropic) treatment of cell e.s.d.'s is used for estimating e.s.d.'s involving l.s. planes.
Refinement. Refinement of *F*^2^ against ALL reflections. The weighted *R*-factor *wR* and goodness of fit *S* are based on *F*^2^, conventional *R*-factors *R* are based on *F*, with *F* set to zero for negative *F*^2^. The threshold expression of *F*^2^ > σ(*F*^2^) is used only for calculating *R*-factors(gt) *etc*. and is not relevant to the choice of reflections for refinement. *R*-factors based on *F*^2^ are statistically about twice as large as those based on *F*, and *R*- factors based on ALL data will be even larger.

### Fractional atomic coordinates and isotropic or equivalent isotropic displacement parameters (Å^2^)

**Table:**

	*x*	*y*	*z*	*U*_iso_*/*U*_eq_	
C1	0.34222 (19)	0.8581 (2)	0.10826 (8)	0.0475 (4)	
C2	0.2573 (3)	0.9698 (3)	0.13547 (12)	0.0688 (6)	
H2	0.2543	0.9701	0.1827	0.083*	
C3	0.1749 (3)	1.0837 (3)	0.09239 (19)	0.0876 (8)	
H3	0.1173	1.1596	0.1109	0.105*	
C4	0.1793 (3)	1.0828 (3)	0.02504 (18)	0.0858 (8)	
H4	0.1241	1.1593	−0.0029	0.103*	
C5	0.2639 (2)	0.9713 (2)	−0.00569 (11)	0.0634 (5)	
C6	0.2687 (4)	0.9715 (4)	−0.07762 (13)	0.0910 (9)	
H6	0.2122	1.0468	−0.1058	0.109*	
C7	0.3515 (4)	0.8673 (4)	−0.10559 (13)	0.1015 (12)	
H7	0.3537	0.8711	−0.1529	0.122*	
C8	0.4342 (4)	0.7536 (3)	−0.06564 (14)	0.0872 (9)	
H8	0.4913	0.6808	−0.0863	0.105*	
C9	0.4342 (2)	0.7451 (2)	0.00414 (11)	0.0619 (5)	
H9	0.4904	0.6666	0.0303	0.074*	
C10	0.34948 (19)	0.85482 (19)	0.03643 (8)	0.0458 (4)	
C11	0.21070 (19)	0.52112 (19)	0.14424 (9)	0.0466 (4)	
C12	0.0822 (2)	0.5617 (2)	0.09445 (10)	0.0595 (5)	
H12	0.0949	0.6176	0.0553	0.071*	
C13	−0.0674 (2)	0.5150 (3)	0.10555 (12)	0.0696 (6)	
H13	−0.1582	0.5414	0.0734	0.084*	
C14	−0.0850 (2)	0.4309 (2)	0.16279 (11)	0.0637 (5)	
H14	−0.1874	0.4025	0.1692	0.076*	
C15	0.0471 (2)	0.3876 (2)	0.21112 (10)	0.0561 (4)	
C16	0.1979 (2)	0.4357 (2)	0.20153 (9)	0.0511 (4)	
H16	0.2890	0.4101	0.2336	0.061*	
O1	0.36734 (15)	0.56180 (15)	0.13433 (7)	0.0580 (3)	
O2	0.3993 (2)	0.7447 (2)	0.23185 (7)	0.0915 (6)	
O3	0.60456 (18)	0.7106 (2)	0.15880 (10)	0.0903 (6)	
N1	0.0316 (3)	0.3014 (3)	0.26868 (11)	0.0868 (6)	
H1A	−0.0621	0.2733	0.2752	0.104*	
H1B	0.1156	0.2758	0.2981	0.104*	
S1	0.44040 (6)	0.72128 (7)	0.16539 (2)	0.06166 (18)	

### Atomic displacement parameters (Å^2^)

**Table:**

	*U*^11^	*U*^22^	*U*^33^	*U*^12^	*U*^13^	*U*^23^
C1	0.0464 (8)	0.0490 (9)	0.0497 (8)	−0.0131 (7)	0.0153 (6)	−0.0086 (7)
C2	0.0679 (12)	0.0677 (13)	0.0789 (13)	−0.0188 (10)	0.0346 (10)	−0.0268 (11)
C3	0.0697 (14)	0.0536 (13)	0.144 (3)	0.0011 (10)	0.0312 (15)	−0.0233 (15)
C4	0.0699 (14)	0.0511 (12)	0.130 (2)	−0.0040 (10)	0.0007 (14)	0.0086 (14)
C5	0.0621 (11)	0.0556 (11)	0.0683 (12)	−0.0236 (9)	0.0003 (9)	0.0073 (9)
C6	0.1060 (19)	0.0930 (19)	0.0634 (13)	−0.0531 (16)	−0.0135 (13)	0.0241 (13)
C7	0.136 (3)	0.118 (2)	0.0525 (13)	−0.082 (2)	0.0230 (15)	−0.0135 (15)
C8	0.1037 (19)	0.0958 (19)	0.0737 (14)	−0.0525 (16)	0.0469 (14)	−0.0426 (14)
C9	0.0619 (11)	0.0660 (12)	0.0638 (11)	−0.0206 (9)	0.0276 (9)	−0.0226 (9)
C10	0.0451 (8)	0.0451 (8)	0.0482 (8)	−0.0160 (6)	0.0112 (6)	−0.0062 (6)
C11	0.0461 (8)	0.0416 (8)	0.0523 (9)	−0.0039 (6)	0.0095 (6)	−0.0014 (7)
C12	0.0603 (10)	0.0606 (11)	0.0536 (10)	−0.0068 (9)	−0.0006 (8)	0.0050 (8)
C13	0.0519 (10)	0.0769 (14)	0.0725 (13)	−0.0074 (9)	−0.0091 (9)	−0.0047 (11)
C14	0.0522 (10)	0.0598 (11)	0.0798 (13)	−0.0175 (8)	0.0139 (9)	−0.0150 (10)
C15	0.0643 (10)	0.0438 (9)	0.0640 (11)	−0.0099 (8)	0.0214 (9)	−0.0058 (8)
C16	0.0524 (9)	0.0452 (9)	0.0546 (9)	−0.0002 (7)	0.0068 (7)	0.0035 (7)
O1	0.0501 (6)	0.0556 (7)	0.0704 (8)	−0.0023 (5)	0.0165 (6)	0.0074 (6)
O2	0.1164 (14)	0.1138 (14)	0.0419 (7)	−0.0494 (11)	0.0078 (8)	−0.0014 (8)
O3	0.0456 (8)	0.1086 (14)	0.1099 (14)	−0.0144 (8)	−0.0046 (8)	0.0307 (11)
N1	0.0905 (14)	0.0891 (15)	0.0865 (14)	−0.0159 (11)	0.0316 (11)	0.0238 (11)
S1	0.0536 (3)	0.0762 (4)	0.0524 (3)	−0.0199 (2)	0.00177 (19)	0.0072 (2)

### Geometric parameters (Å, °)

**Table:**

C1—C2	1.370 (3)	C9—H9	0.9300
C1—C10	1.420 (2)	C11—C16	1.365 (2)
C1—S1	1.7368 (19)	C11—C12	1.370 (2)
C2—C3	1.401 (4)	C11—O1	1.4173 (19)
C2—H2	0.9300	C12—C13	1.382 (3)
C3—C4	1.327 (4)	C12—H12	0.9300
C3—H3	0.9300	C13—C14	1.368 (3)
C4—C5	1.401 (4)	C13—H13	0.9300
C4—H4	0.9300	C14—C15	1.381 (3)
C5—C6	1.417 (3)	C14—H14	0.9300
C5—C10	1.418 (3)	C15—N1	1.378 (3)
C6—C7	1.321 (5)	C15—C16	1.386 (2)
C6—H6	0.9300	C16—H16	0.9300
C7—C8	1.370 (5)	O1—S1	1.5905 (14)
C7—H7	0.9300	O2—S1	1.4212 (16)
C8—C9	1.369 (3)	O3—S1	1.4199 (16)
C8—H8	0.9300	N1—H1A	0.8600
C9—C10	1.408 (3)	N1—H1B	0.8600
			
C2—C1—C10	121.24 (18)	C5—C10—C1	117.02 (17)
C2—C1—S1	117.12 (15)	C16—C11—C12	123.72 (16)
C10—C1—S1	121.63 (13)	C16—C11—O1	117.47 (15)
C1—C2—C3	120.2 (2)	C12—C11—O1	118.71 (16)
C1—C2—H2	119.9	C11—C12—C13	116.40 (18)
C3—C2—H2	119.9	C11—C12—H12	121.8
C4—C3—C2	119.7 (2)	C13—C12—H12	121.8
C4—C3—H3	120.2	C14—C13—C12	121.48 (18)
C2—C3—H3	120.2	C14—C13—H13	119.3
C3—C4—C5	122.6 (2)	C12—C13—H13	119.3
C3—C4—H4	118.7	C13—C14—C15	120.92 (18)
C5—C4—H4	118.7	C13—C14—H14	119.5
C4—C5—C6	122.3 (3)	C15—C14—H14	119.5
C4—C5—C10	119.2 (2)	N1—C15—C14	121.64 (19)
C6—C5—C10	118.5 (2)	N1—C15—C16	119.91 (19)
C7—C6—C5	121.6 (3)	C14—C15—C16	118.43 (17)
C7—C6—H6	119.2	C11—C16—C15	119.01 (16)
C5—C6—H6	119.2	C11—C16—H16	120.5
C6—C7—C8	120.6 (2)	C15—C16—H16	120.5
C6—C7—H7	119.7	C11—O1—S1	118.20 (11)
C8—C7—H7	119.7	C15—N1—H1A	120.0
C9—C8—C7	121.2 (3)	C15—N1—H1B	120.0
C9—C8—H8	119.4	H1A—N1—H1B	120.0
C7—C8—H8	119.4	O3—S1—O2	119.75 (11)
C8—C9—C10	120.2 (2)	O3—S1—O1	103.16 (10)
C8—C9—H9	119.9	O2—S1—O1	109.45 (9)
C10—C9—H9	119.9	O3—S1—C1	110.36 (9)
C9—C10—C5	117.90 (18)	O2—S1—C1	108.99 (11)
C9—C10—C1	125.09 (18)	O1—S1—C1	103.85 (7)
			
C10—C1—C2—C3	0.2 (3)	C16—C11—C12—C13	1.8 (3)
S1—C1—C2—C3	−179.87 (16)	O1—C11—C12—C13	178.00 (17)
C1—C2—C3—C4	0.1 (3)	C11—C12—C13—C14	−0.9 (3)
C2—C3—C4—C5	0.1 (4)	C12—C13—C14—C15	−1.0 (3)
C3—C4—C5—C6	−179.7 (2)	C13—C14—C15—N1	−179.3 (2)
C3—C4—C5—C10	−0.5 (3)	C13—C14—C15—C16	2.1 (3)
C4—C5—C6—C7	178.7 (2)	C12—C11—C16—C15	−0.7 (3)
C10—C5—C6—C7	−0.6 (3)	O1—C11—C16—C15	−176.96 (15)
C5—C6—C7—C8	0.9 (4)	N1—C15—C16—C11	−179.90 (18)
C6—C7—C8—C9	−0.4 (4)	C14—C15—C16—C11	−1.3 (3)
C7—C8—C9—C10	−0.5 (3)	C16—C11—O1—S1	−91.61 (17)
C8—C9—C10—C5	0.8 (3)	C12—C11—O1—S1	91.97 (18)
C8—C9—C10—C1	−179.44 (16)	C11—O1—S1—O3	168.69 (12)
C4—C5—C10—C9	−179.52 (17)	C11—O1—S1—O2	40.15 (16)
C6—C5—C10—C9	−0.3 (2)	C11—O1—S1—C1	−76.12 (13)
C4—C5—C10—C1	0.7 (2)	C2—C1—S1—O3	−127.86 (16)
C6—C5—C10—C1	179.93 (16)	C10—C1—S1—O3	52.09 (16)
C2—C1—C10—C9	179.68 (17)	C2—C1—S1—O2	5.58 (17)
S1—C1—C10—C9	−0.3 (2)	C10—C1—S1—O2	−174.48 (13)
C2—C1—C10—C5	−0.6 (2)	C2—C1—S1—O1	122.16 (14)
S1—C1—C10—C5	179.50 (12)	C10—C1—S1—O1	−57.89 (14)

### Hydrogen-bond geometry (Å, °)

**Table:**

*D*—H···*A*	*D*—H	H···*A*	*D*···*A*	*D*—H···*A*
C2—H2···O2	0.93	2.41	2.829 (3)	107
C9—H9···O3	0.93	2.56	3.127 (3)	120
N1—H1B···O3^i^	0.86	2.43	3.246 (3)	158
C7—H7···O2^ii^	0.93	2.56	3.422 (3)	154

Symmetry codes: (i) −*x*+1, *y*−1/2, −*z*+1/2; (ii) *x*, −*y*+3/2, *z*−1/2.
